# Leucine‐rich repeat containing protein LRRC8A is essential for swelling‐activated Cl^−^ currents and embryonic development in zebrafish

**DOI:** 10.14814/phy2.12940

**Published:** 2016-09-28

**Authors:** Toshiki Yamada, Robert Wondergem, Rebecca Morrison, Viravuth P. Yin, Kevin Strange

**Affiliations:** ^1^ MDI Biological LaboratoryDavis Center for Regenerative Biology and MedicineSalisbury CoveMaine; ^2^ Department of Biomedical SciencesJames H. Quillen College of MedicineEast Tennessee State UniversityJohnson CityTennessee

**Keywords:** Cell swelling, cell volume regulation, chloride channel, organic anion channel

## Abstract

A volume‐regulated anion channel (VRAC) has been electrophysiologically characterized in innumerable mammalian cell types. VRAC is activated by cell swelling and mediates the volume regulatory efflux of Cl^−^ and small organic solutes from cells. Two groups recently identified the mammalian leucine‐rich repeat containing protein LRRC8A as an essential VRAC component. *LRRC8A* must be coexpressed with at least one of the other four members of this gene family, *LRRC8B‐E*, to reconstitute VRAC activity in *LRRC8* ^*−/−*^ cells. *LRRC8* genes likely arose with the origin of chordates. We identified *LRRC8A* and *LRRC8C‐E* orthologs in the zebrafish genome and demonstrate that zebrafish embryo cells and differentiated adult cell types express a swelling‐activated Cl^−^ current indistinguishable from mammalian VRAC currents. Embryo cell VRAC currents are virtually eliminated by morpholino knockdown of the zebrafish *LRRC8A* ortholog *lrrc8aa*. VRAC activity is fully reconstituted in *LRRC8* ^*−/−*^ human cells by coexpression of zebrafish *lrrc8aa* and human *LRRC8C* cDNAs. *lrrc8aa* expression varies during zebrafish embryogenesis and *lrrc8aa* knockdown causes pericardial edema and defects in trunk elongation and somatogenesis. Our studies provide confirmation of the importance of *LRRC8A* in VRAC activity and establish the zebrafish as a model system for characterizing the molecular regulation and physiological roles of VRAC and LRRC8 proteins.

## Introduction

A swelling‐activated, outwardly rectifying anion current has been identified and studied in innumerable cell types and may be expressed ubiquitously in vertebrates. The channel responsible for this current has been referred to by several names, including volume‐regulated anion channel or VRAC. VRAC mediates the efflux of Cl^−^ and small organic solutes from cells required for regulatory volume decrease (RVD) following cell swelling (McManus et al. 1995; Strange et al. 1996). Indirect evidence suggests that the channel may also play roles in other important physiological processes, including angiogenesis, apoptosis, cell proliferation, and migration and intercellular communication (reviewed in Mulligan and MacVicar 2006; Hoffmann et al. 2009; Duran et al. 2010; Pedersen et al. 2015).

Volume‐regulated anion channel activity was first identified in 1988 in human T lymphocytes (Cahalan and Lewis 1988) and human intestine cells (Hazama and Okada 1988). Efforts to identify the gene(s) encoding VRAC have been extensive since the early 1990s. However, previous gene candidates who have been identified either failed experimental confirmation or lacked properties that fully recapitulate the channel (reviewed in Nilius and Droogmans 2003; Pedersen et al. 2015; Stauber 2015).

Molecular understanding of VRAC was greatly complicated by technical limitations. Expression cloning and heterologous expression of an ion channel requires cell systems with no or minimal background expression of the same or related channels. VRAC is expressed in virtually all vertebrate cell types examined. Invertebrate cells may not express VRAC per se, but certainly express other volume‐sensitive anion channels (Villaz et al. 1995; Rutledge et al. 2001; Chien and Hartzell 2007; Coulon et al. 2008).

The development of RNA interference technology opened the door for genome‐wide reverse genetic screening to identify genes underlying diverse physiological processes (Mohr et al. 2014). Two groups recently utilized this approach in mammalian cells to identify what appear to be bona fide molecular components of VRAC. In independent studies, Voss et al. (2014) and Qiu et al. (2014) both demonstrated that silencing of the human *LRRC8A* (leucine‐rich repeat containing 8A) gene expression eliminates VRAC activity. *LRRC8A* must be coexpressed with one of the other four *LRRC8* gene family members, *LRRC8B‐E*, to reconstitute VRAC activity and to establish anionic substrate specificity (Voss et al. 2014; Syeda et al. 2016).

The goal of the current study was to further validate the role of *LRRC8A* in VRAC activity. We demonstrate that zebrafish embryo cells and various differentiated adult cell types express a typical VRAC current. In embryo cells, VRAC activity is virtually eliminated by knockdown of the zebrafish *LRRC8A* ortholog, *lrrc8aa*. VRAC activity is fully reconstituted in *LRRC8* ^*−/−*^ human cells by coexpression of zebrafish *lrrc8aa* and human *LRRC8C* cDNAs. *lrrc8aa* mRNA levels vary widely during early development and morpholino knockdown of *lrrc8aa* expression disrupts zebrafish embryogenesis. Our studies confirm the importance of *LRRC8A* in VRAC activity and establish the zebrafish as a model system for characterizing the molecular regulation and physiological roles of VRAC and LRRC8 proteins.

## Materials and Methods

### Isolation of differentiated adult zebrafish cells

Adult zebrafish hearts were dissected from wild‐type Ekkwill (EK) fish anesthetized with 2‐phenoxyethanol. Three hearts were pooled in an isolation buffer containing 100 mmol/L NaCl, 10 mmol/L KCl, 1.2 mmol/L KH_2_PO_4_, 4 mmol/L MgSO_4_, 50 mmol/L taurine, 20 mmol/L glucose, and 10 mmol/L HEPES (pH 7.40, 290 mOsm). Ventricles were dissected from atria and outflow tracts, rinsed to remove blood cells, and then incubated with gentle rocking in isolation buffer containing 1 mg/mL type 1 collagenase (Sigma‐Aldrich, St. Louis, MO). After 10 min, ventricles were cut into 4–5 pieces with a razor, incubated for 30 min in isolation buffer with collagenase and transferred into a plastic Petri dish where the tissue was minced to dissociate cells by pressing with a glass coverslip. Dissociated cells were incubated for 5–10 min in isolation buffer and then centrifuged at 200 × ***g*** for 2 min. The pellet was rinsed with an isolation buffer containing 0.01% albumin (Sigma‐Aldrich). Isolated cardiomyocytes were maintained in isolation buffer with albumin at room temperature and used within 3 h.

Adult zebrafish kidneys were dissected from EK fish anesthetized with 2‐phenoxyethanol. Isolated kidneys were rinsed in isolation buffer, transferred to 0.25% trypsin‐EDTA solution (Sigma‐Aldrich) and incubated for 15 min with gentle rotation. After incubation, the kidneys were mechanically dissociated with gentle trituration. Trypsinization was stopped by adding isolation buffer containing 0.01% albumin. Dissociated cells were pelleted by centrifugation at 200 × ***g***, rinsed once with isolation buffer containing 0.01% albumin and then maintained in this medium at room temperature for no more than 3 h before patch clamp measurements were performed.

Blood cells were isolated by rinsing dissected ventricles in isolation buffer and pipetting the diluted blood directly onto 12 mm diameter poly‐l‐lysine‐coated cover slips. After allowing blood cells to settle, the chips were rinsed with isolation buffer and maintained in this medium at room temperature for no more than 3 h before patch clamp measurements were performed.

### Isolation of zebrafish embryo cells

Fifteen 24–48 hours postfertilization (hpf) wild‐type EK zebrafish embryos were dechorionated by incubation in 8% Hank's Balanced Salt Solution (HBSS; Lonza Biologics, Inc., Portsmouth, NH) containing 0.1 mmol/L CaCl_2_ and 0.1 mmol/L MgSO_4_ and 1 mg/ml Type XIV *Streptomyces griseus* protease (Sigma‐Aldrich). After 3 min, embryos were rinsed three times in the same solution without protease and then incubated for 1 min with gentle trituration in 0.25% trypsin‐EDTA solution (GE Healthcare Life Sciences, Logan UT) diluted 1:5 with Ca^2+^‐ and Mg^2+^‐free 80% HBSS. Trypsinization was stopped by adding 80% HBSS stop solution containing 1 mmol/L CaCl_2_, 1 mmol/L MgSO_4_ and 0.01% albumin (Sigma‐Aldrich). Dissociated cells were pelleted by centrifugation at 200 × ***g***, rinsed once with the HBSS stop solution and then maintained in this medium at room temperature for no more than 3 h before patch clamp measurements were performed.

### Patch clamp of isolated zebrafish cells

Dissociated zebrafish adult and embryo cells were placed on a poly‐l‐lysine‐coated coverslip and patch clamped, using a bath solution containing 90 mmol/L NMDG‐Cl, 5 mmol/L MgSO_4_, 1 mmol/L CaCl_2_, 12 mmol/L HEPES, 8 mmol/L Tris, 5 mmol/L glucose, 90 mmol/L sucrose, and 2 mmol/L glutamine (pH 7.4, 300 mOsm) and a pipette solution containing 80 mmol/L or 98 mmol/L NMDG‐Cl or CsCl, 2 mmol/L MgSO_4_, 20 mmol/L HEPES, 1 mmol/L EGTA, 2 mmol/L ATP, 0.5 mmol/L GTP, and 85 mmol/L or 60 mmol/L sucrose (pH 7.2, 275 mOsm). Cells were swollen by exposure to a 250 mOsm bath solution. Osmolality was measured by freezing point depression (Osmette II; Precision Systems, Inc., Natick, MA), and adjusted by addition or removal of sucrose.

Patch electrodes were pulled from 1.5 mm outer diameter silanized borosilicate microhematocrit tubes; electrode resistance ranged from 3 to 8 MΩ. Currents were measured with an Axopatch 200A (Axon Instruments, Foster City, CA) patch clamp amplifier. Electrical connections to the patch clamp amplifier were made using Ag/AgCl wires and 3 mol/L KCl/agar bridges. Data acquisition and analysis were performed, using pClamp 10 software (Axon Instruments). Whole cell currents were elicited using voltage ramp and step protocols. Membrane potential was held at 0 mV.

### Heterologous expression and patch clamping of *LRRC8* homologs

Human colon cancer HCT116 cells in which the five *LRRC8* genes were disrupted by genome editing (i.e., *LRRC8* ^*−/−*^) were a kind gift from Dr. Thomas Jentsch. *LRRC8* ^*−/−*^ cells were cultured in 15 mm diameter wells using McCoy's 5A medium containing 10% fetal bovine serum, 50 *μ*g/ml penicillin and 50 *μ*g/ml streptomycin. At ~70% confluence, cells were transfected using Turbofectin 8.0 (OriGene Technologies, Rockville, MD) with 0.125 *μ*g GFP and either 0.25 *μ*g human *LRRC8A* and 0.25 *μ*g human *LRRC8C* cDNAs or 0.25 *μ*g zebrafish *lrrc8aa* and 0.25 *μ*g human *LRRC8C* cDNAs. Zebrafish *lrrc8aa* cDNA cloned into pME18S‐FL3 and human *LRRC8A* and *LRRC8C* cDNAs cloned into pCMV6 were purchased from GE Dharmacon (Lafayette, CO; catalog number MDR1734‐202795972) and OriGene Technologies (catalog numbers RC226180 and RC222603), respectively.

Following transfection, cells were incubated at 37°C for 24–36 h. Approximately 2 h prior to patch clamp experiments, cells were detached from growth plates by exposure to 0.25% trypsin‐EDTA (GE Healthcare Life Sciences) for 2 min. Detached cells were suspended in culture medium, pelleted by centrifugation, resuspended in fresh culture medium and then plated onto 12 mm diameter poly‐l‐lysine‐coated cover slips for patch clamping.

Transfected cells were identified by GFP fluorescence and patch clamped, using a bath solution containing 90 mmol/L NMDG‐Cl, 5 mmol/L MgSO_4_, 1 mmol/L CaCl_2_, 12 mmol/L HEPES, 8 mmol/L Tris, 5 mmol/L glucose, 90 mmol/L sucrose and 2 mmol/L glutamine (pH 7.4, 300 mOsm), and a pipette solution containing 116 mmol/L NMDG‐Cl, 2 mmol/L MgSO_4_, 20 mmol/L HEPES, 6 mmol/L CsOH, 1 mmol/L EGTA, 2 mmol/L ATP, 0.5 mmol/L GTP, and 10 mmol/L sucrose (pH 7.2, 275 mOsm). Cells were swollen and shrunken by exposure to 250 and 400 mOsm bath solutions, respectively. Osmolality was adjusted by addition or removal of sucrose. Patch clamp methods were similar to those described for adult and embryonic zebrafish cells.

### Measurement of relative anion permeabilities (*P*_x_/*P*_Cl_)

Relative anion permeabilities were measured in zebrafish and transfected HCT116 cells from changes in reversal potential using a modified Goldman–Hodgkin–Katz equation (Voss et al. 2014). For these studies, the bath solution contained 70 or 115 mmol/L NaCl with osmolality adjusted by sucrose. Bath NaCl was replaced with either 70 or 115 mmol/L NaSCN, 70 or 115 mmol/L NaI or 200 mmol/L taurine. For the taurine solution, pH was adjusted to 8.2, which gives a calculated concentration of negatively charged taurine of 58 mmol/L. Raising bath pH to 8.2 did not significantly alter the Cl^−^ reversal potential (data not shown). Reversal potentials were corrected for anion‐induced changes in liquid junction potentials.

### Morpholino design and injection

Antisense morpholinos (MO) were designed to either the ATG start site of the *lrrc8aa* gene (NM_001029949.1) (5′‐ACCGCAGCTCAGTGATGGGAATCAT‐3′) to block translation or directed against the exon‐3/intron‐3 splice site (5′‐ACACTATAAACCCAACGCACCTCCA‐3′) (Gene‐Tools, LLC, Philomath, OR) to block mRNA splicing. Scrambled (control) MO or *lrrc8aa* MO were microinjected in a volume of 1 nL into wild‐type EK zebrafish embryos maintained at 28°C. Embryos that failed to progress to the 1000‐cell stage by 8 hpf were considered to be unfertilized and were discarded.

### Quantification of *lrrc8aa* expression in zebrafish embryos

Wild‐type EK zebrafish embryos were staged at 28°C from the onset of fertilization until 48 hpf. Fifty to 70 embryos corresponding to 1000‐cell, shield, tailbud, 15‐somites, 24 and 48 hpf stages were collected into 1.5 mL micro‐centrifuge tubes. Total RNA was extracted, using TriZol Reagent (ThermoFisher Scientific, Waltham, MA). cDNA synthesis was performed using a ProtoScript II First Strand cDNA Synthesis Kit (New England BioLabs, Ipswich, MA). *lrrc8aa* was quantified by quantitative real‐time polymerase chain reaction (qRT‐PCR), using Brilliant III SYBR green (Agilent Technologies, Santa Clara, CA) and a LightCycler 480 (Roche Diagnostics, Corp., Indianapolis, IN). Normalization and data analysis were performed as previously described (Yin et al. 2008).

### Characterization of *lrrc8aa* knockdown on embryogenesis

The effect of *lrrc8aa* knockdown by morpholino injection was assessed by measuring larval trunk length from the end of the yolk sac to the end of the tail. Normal, moderate and severe phenotypes were defined as trunks lengths ≥95%, 50–95% and <50%, respectively, of the mean trunk length measured in larvae developing from embryos injected with a control morpholino. Penetrance is defined as the proportion of larvae expressing a normal, moderate or severe phenotype.

### Statistical analysis

All data are presented as means ± SE. Statistical significance was determined, using Student's two tailed *t*‐test when two means were compared. *P* values of ≤0.05 were taken to indicate statistical significance.

## Results

**Figure 1:**
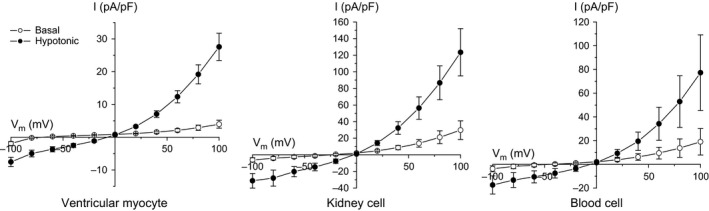
Current‐to‐voltage relationships of swelling‐activated anion currents in various cell types from adult zebrafish. Plots show basal current and peak current observed 6 min after induction of swelling by exposure of cells to a 250 mOsm. Currents were recorded from dissociated ventricular myocytes and kidney cells and unidentified blood cells. Whole cell currents were evoked by ramping membrane potential from −100 to +100 mV over 200 msec. Cells were held at 0 mV for 5 sec between voltage ramps. Values are means ± SE (*n* = 4–7).

To determine whether zebrafish express a VRAC‐like current, we patch clamped ventricular myocytes, kidney cells, and blood cells isolated from adult zebrafish. All three cell types expressed an outwardly rectifying, swelling‐activated anion current (Fig. 1).

**Figure 2:**
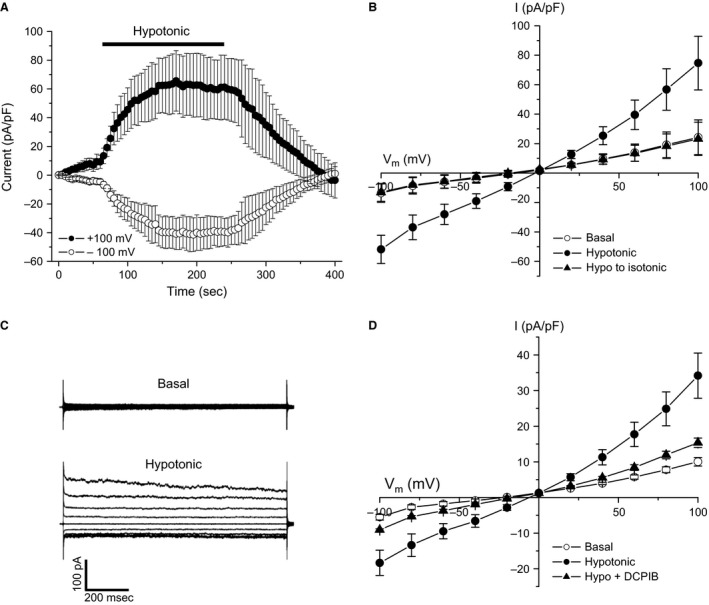
Characteristics of swelling‐activated anion currents in zebrafish embryo cells. (A) Time course of swelling‐induced activation and shrinkage‐induced inactivation of whole cell Cl^−^ currents. Cells were swollen by exposure to a 250 mOsm bath solution and then shrunken by returning to an isotonic 300 mOsm bath. Currents were evoked by ramping membrane potential from −100 to +100 mV over 200 msec. Cells were held at 0 mV for 5 sec between voltage ramps. For each cell, basal anion currents measured during the first 30 sec after obtaining whole cell access were averaged and subtracted from all current recordings. Values are means ± SE (*n* = 3). (B) Current‐to‐voltage relationships of basal current, peak current observed 2.5 min after induction of swelling by exposure of cells to a 250 mOsm bath solution and current measured 2 min after shrinking swollen cells by returning them to isotonic 300 mOsm bath solution. Currents were evoked as described in (A). Values are means ± SE (*n* = 3). (C) Representative whole cell current traces of basal and swelling‐activated anion currents. Currents were evoked by voltage clamping cells from −120 to +120 mV for 1 sec in 30 mV increments from a holding potential of 0 mV. Each test pulse was followed by a 2‐sec recovery period at 0 mV. (D) Effect of 10 *μ*mol/L DCPIB on swelling‐activated whole cell anion current. DCPIB was added to the bath 2 min after cell swelling was induced by exposure to a 250 mOsm bath solution. Inhibitory effect of DCPIB was complete within 2 min after adding the drug to the bath solution. Currents were evoked as described in (A). Values are means ± SE (*n* = 3).

Cells isolated from 24 to 48 h stage embryos also expressed a swelling‐activated anion current (Fig. 2A). Because gene expression can be readily knocked down in embryo cells, using morpholino strategies, we characterized the swelling‐activated anion current in this cell type in greater detail. The current activated rapidly in response to cell swelling and was inactivated by returning cells to an isotonic bath solution (Fig. 2A). Whole cell current exhibited modest outward rectification (Fig. 2B), inactivated in a time‐dependent fashion at strongly depolarized membrane potentials (Fig. 2C) and was fully inhibited by 10 μmol/L DCPIB (Fig. 2D), a reportedly selective inhibitor of VRAC (Decher et al. 2001). Relative anion permeability (*P*_x_/*P*_Cl_) of the conductance showed a selectivity sequence of SCN^−^ > I^−^ > Cl^−^ > taurine^−^ (Table 1). The characteristics of the zebrafish embryo cell swelling‐activated anion current recapitulate those of VRAC currents identified in numerous other vertebrate cells types (Mulligan and MacVicar 2006; Hoffmann et al. 2009; Duran et al. 2010; Pedersen et al. 2015).

**Table 1:** Relative anion permeability of swelling‐activated anion conductances in zebrafish embryo cells and *LRRC8* ^*−/−*^ HCT116 cells transfected with *LRRC8* cDNAs

Cell type	Anion	*E*_rev_ (mV)	*P*_x_/*P*_Cl_
Zebrafish embryo cells	115 mmol/L Cl^−^	−9.1 ± 1.6	
	115 mmol/L SCN^−^	−21.4 ± 3.9	1.8 ± 0.2
	115 mmol/L I^−^	−16.5 ± 2.0	1.5 ± 0.1
	58 mmol/L taurine^−^	27.2 ± 4.5	0.19 ± 0.04
*LRRC8* ^*−/−*^ HCT116 cells transfected with human *LRRC8A* and human *LRRC8C* cDNAs	70 mmol/L Cl^−^	12.0 ± 0.9	
	70 mmol/L SCN^−^	−2.8 ± 1.6	1.8 ± 0.07
	70 mmol/L I^−^	4.4 ± 0.9	1.3 ± 0.05
	58 mmol/L taurine^−^	52.1 ± 2.3	0.21 ± 0.02
*LRRC8* ^*−/−*^ HCT116 cells transfected with zebrafish *lrrc8aa* and human *LRRC8C* cDNAs	70 mmol/L Cl^−^	12.0 ± 1.1	
	70 mmol/L SCN^−^	−0.9 ± 1.4	1.7 ± 0.05
	70 mmol/L I^−^	2.4 ± 0.9	1.5 ± 0.05
	58 mmol/L taurine^−^	50.4 ± 1.7	0.23 ± 0.01

Values are means ± SE (*n* = 4–6).

**Figure 3:**
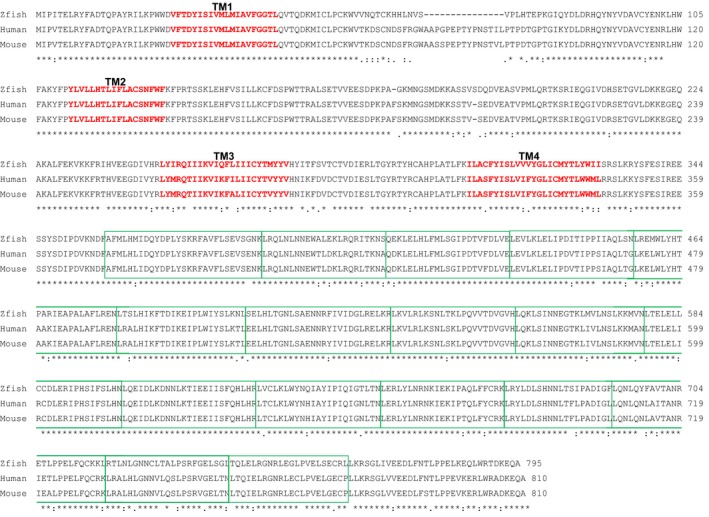
Alignment of predicted zebrafish Lrrc8aa and human and mouse LRRC8A amino acid sequences. Predicted transmembrane (TM) domains are shown in red. Green boxes outline 17 predicted (http://www.uniprot.org/uniprot/Q8IWT6) leucine‐rich repeat domains located on the cytoplasmic carboxy‐terminus.

The zebrafish genome contains two *LRRC8A* gene orthologs, *lrrc8aa,* and *lrrc8ab* that share 87% identity with human *LRRC8A*. These two genes likely arose through a whole genome duplication event that occurred in teleost fish ca. 350 million years ago (Glasauer and Neuhauss 2014). *lrrc8aa* transcripts are expressed much more abundantly than transcripts of *lrrc8ab* (http://www.ensembl.org/index.html). We therefore focused our studies on the *lrrc8aa* ortholog. Figure 3 shows an alignment of zebrafish Lrrc8aa and human and mouse LRRC8A predicted amino acid sequences.

**Figure 4:**
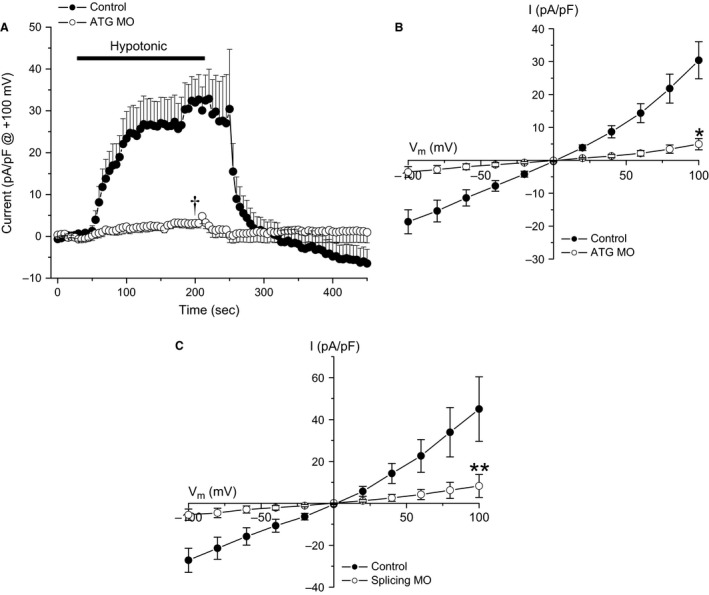
Inhibition of embryo cell swelling activated Cl^−^ currents with translation (ATG) and splice blocking (Splicing) morpholino (MO) knockdown of *lrrc8aa*. (A, B) Time course and current‐to‐voltage relationships of swelling induced Cl^−^ currents in embryo cells injected with control or ATG MO. For each cell in (A), basal anion currents measured during the first 30 sec after obtaining whole cell access were averaged and subtracted from all current recordings. Values are means ± SE (*n* = 6–8). ^†^ *P* < 0.0004 compared to control. *P < 0.003 compared to control at all voltages. (C) Current‐to‐voltage relationships of swelling‐activated Cl^−^ currents in embryo cells injected with control or Splicing MO. Values are means ± SE (*n* = 5–7). **P < 0.03 for all voltages except +20 mV. Cell swelling was induced in all experiments by exposure to a 250 mOsm bath solution and currents were evoked by ramping membrane potential from −100 to +100 mV over 200 msec. Cells were held at 0 mV for 5 sec between voltage ramps.

To determine whether zebrafish Lrrc8aa is required for swelling‐activated anion currents activity in embryo cells, we knocked down *lrrc8aa* expression, using a translation (ATG) blocking morpholino. One‐cell stage embryos were injected with the morpholino and embryo cells 24–48 hpf were patch clamped. As shown in Figures 4A and B, morpholino knockdown of zebrafish *lrrc8aa* virtually eliminated swelling‐activated anion currents in embryo cells.

To further test the role of zebrafish Lrrc8aa in swelling‐activated anion current activity, we generated a splice blocking morpholino to inhibit *lrrc8aa* pre‐mRNA processing. Knockdown of *lrrc8aa* expression by the splice blocking morpholino also dramactically reduced swelling‐activated anion currents in 24–48 hpf embryo cells (Fig. 4C).

**Figure 5:**
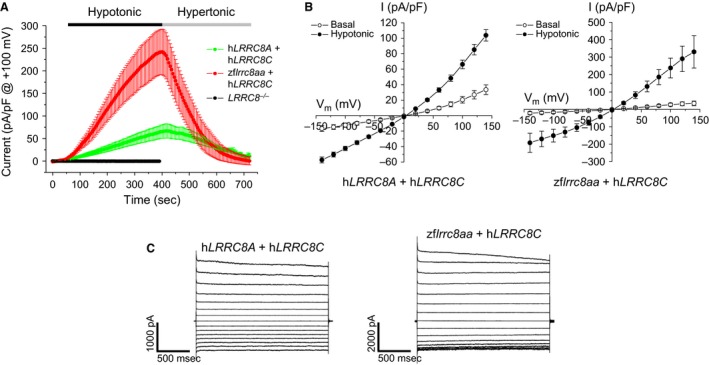
Expression of *LRRC8* cDNAs in *LRRC8* ^*−/−*^ HCT116 cells. (A) Time course of swelling‐induced activation and shrinkage‐induced inactivation of whole cell Cl^−^ currents. Cells were swollen by exposure to a 250 mOsm bath solution and then shrunken by returning to a hypertonic 400 mOsm bath. Currents were evoked by ramping membrane potential from −100 to +100 mV over 200 msec. Cells were held at 0 mV for 5 sec between voltage ramps. Values are means ± SE (*n* = 3–8). (B) Current‐to‐voltage relationships of basal and peak swelling‐activated Cl^−^ currents. Values are means ± SE (*n* = 4). (C) Representative whole cell current traces of swelling‐activated anion currents. Currents in (B) and (C) were evoked by voltage clamping cells from −140 to +140 mV for 2 sec in 20 mV increments from a holding potential of 0 mV. Each test pulse was followed by a 3‐sec recovery period at 0 mV.

As noted earlier, human *LRRC8A* is essential for VRAC activity (Qiu et al. 2014; Voss et al. 2014). Four other gene family members, *LRRC8B‐E*, are typically present in mammalian genomes (Abascal and Zardoya 2012). VRAC activity requires coexpression of *LRRC8A* with *LRRC8B, C, D,* or *E* (Voss et al. 2014; Syeda et al. 2016). As a further test for the function of zebrafish *lrrc8aa*, we coexpressed it together with human *LRRC8C* in human colon cancer HCT116 cells in which the five *LRRC8* genes were disrupted by genome editing (i.e., *LRRC8* ^*−/−*^). As shown in Figure 5A, VRAC currents were not detected in *LRRC8* ^*−/−*^ cells. Expression of any of the three cDNAs alone did not induce swelling‐activated anion currents (data not shown). However, robust currents were detected in *LRRC8* ^*−/−*^ cells coexpressing either human *LRRC8A* and *LRRC8C* or zebrafish *lrrc8aa* and human *LRRC8C* cDNAs (Fig. 5A). Transfected cells (Fig. 5B) also had basal current activity that was greater than untransfected *LRRC8* ^*−/−*^ cells (data not shown).

Overall, the zebrafish/human chimera current and human current had similar biophysical properties. Relative anion selectivity of the chimera and human conductances were not significantly (*P* > 0.05) different (Table 1). Both conductances showed an anion selectivity sequence of SCN^−^ > I^−^ > Cl^−^ > taurine^−^. This selectivity is similar for VRAC currents measured in countless other cells types (Mulligan and MacVicar 2006; Hoffmann et al. 2009; Duran et al. 2010; Pedersen et al. 2015) as well as currents measured in LRRC8 knockout cells coexpressing LRRC8A and LRRC8C (Voss et al. 2014; Syeda et al. 2016).

The chimera current showed somewhat more rapid voltage‐dependent inactivation compared to the human current (Fig. 5C). Inactivation was quantified as the ratio of the peak current measured immediately after stepping membrane potential to +140 mV and the current measured 2 sec later (i.e., *I*_2 sec_/*I*_peak_). Mean ± SE *I*_2 sec_/*I*_peak_ for the chimera and human currents were 0.78 ± 0.03 and 0.91 ± 0.02 (*n* = 5–7), respectively, and were significantly (*P* < 0.004) different.

The most striking difference between the two currents was peak current amplitude. Chimera VRAC currents were ~3.6‐fold larger than the human currents (Fig. 5A). Both Voss et al. (2014) and Qiu et al. (2014) observed that expression of LRRC8A alone in wild‐type cells suppresses endogenous VRAC activity. This observation suggests that the stoichiometry of LRRC8A with respect to other VRAC components may play an important role in channel function.

The zebrafish and human *LRRC8* cDNAs are cloned into different expression vectors and thus may be expressed at different levels in HCT116 *LRRC8* ^*−/−*^ cells. A simple explanation for the larger chimera VRAC currents then is that the zebrafish *lrrc8aa* cDNA is expressed at lower levels than the human clone. To test this possibility, we monitored the effect of expression of different ratios of *LRRC8A* and *LRRC8C*. Reducing the amount of transfected human *LRRC8A* cDNA by fivefold increased mean ± SE peak swelling‐activated current from 60 ± 12 pA/pF (*n* = 7) to 253 ± 65 pA/pF (*n* = 6) (*P* < 0.009). When the amount of transfected zebrafish *lrrc8aa* cDNA was increased fivefold, peak swelling‐activated current was reduced from 240 ± 44 pA/pF (*n* = 9) to 94 ± 23 pA/pF (*n* = 5) (*P* < 0.03). These results indicate that the ratio of *LRRC8A* to *LRRC8C* expression plays an important role in channel function and may explain the difference in the amplitudes of chimera and human VRAC currents shown in Figure 5A. Additional and more detailed studies will be required to fully understand this interesting observation.

**Figure 6:**
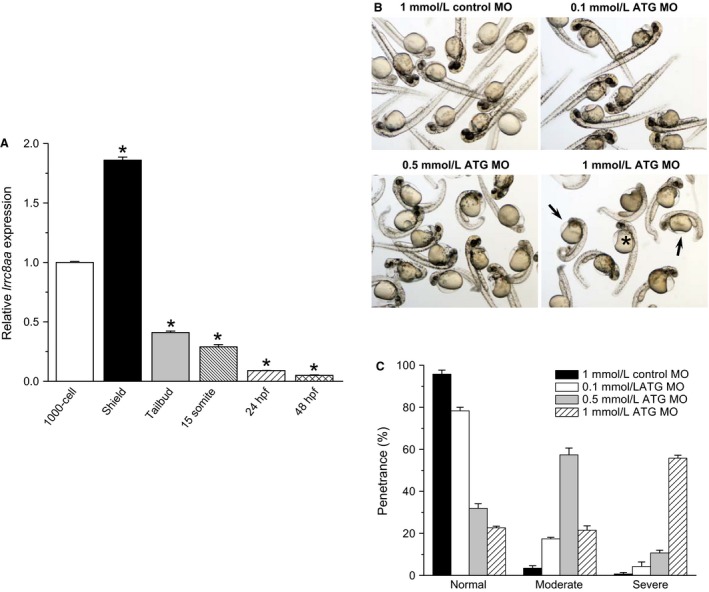
Expression of *lrrc8aa* and effect of *lrrc8aa* knockdown during zebrafish embryogenesis. (A) Relative expression of *lrrc8aa* during various stages of embryonic development. Values are means ± SE (*n* = 3). *P < 0.002 compared to 1000‐cell stage. (B) Brightfield micrographs of 48 hpf larvae developing from one‐cell stage embryos injected with 1 mmol/L control MO or 0.1–1 mmol/L *lrrc8aa* ATG MO. Asterisk and arrows show larvae with moderate and severe phenoptypes, respectively. (C) Penetrance of larval phenotypes. Values are means ± SE (*n* = 3 independent experiments with 185–232 animals).

To begin elucidating the physiological roles of *lrrc8aa*, we quantified expression levels by qPCR during development from the 1000‐cell stage to 48 hpf. When normalized to the 1000‐cell stage, *lrrc8aa* expression increased by 1.86‐fold at the shield stage and then decreased to near undetectable levels by 48 hpf (Fig. 6A). Increased expression of *lrrc8aa* during early embryogenesis coincides with the initiation of zygotic transcription and suggests that this gene may play roles during early morphogenesis.

Given the striking changes in *lrrc8aa* expression during early development, we quantified the effect of gene knockdown on embryogenesis. One‐cell stage embryos were microinjected with 1 mmol/L control morpholino or 0.1–1 mmol/L of the translation blocking (ATG MO) and embryo phenotypes were scored at 48 hpf. *lrrc8aa* knockdown‐induced significant dose‐dependent defects in embryo development (Fig. 6B and C). The most prominent defects observed were pericardial edema and defects in trunk elongation and somatogenesis (Fig. 6B). In contrast, the control morpholino had no obvious effect on developing embryos (Fig. 6B and C).

## Discussion

*LRRC8* genes have been identified in all sequenced vertebrate genomes. These genes likely arose with the origin of chordates and may have resulted from the combination of a pannexin channel with a leucine‐rich repeat domain (Abascal and Zardoya 2012).

Mammalian cells express five *LRRC8* genes (*LRRC8A‐E*). LRRC8 proteins contain four predicted membrane spanning domains and a cytoplasmic carboxy terminus with up to 17 predicted leucine‐rich repeat domains (Fig. 3). Using cultured cells in which various combinations of the five mammalian *LRRC8* genes were knocked out, Voss et al. (2014) and Syeda et al. (2016) have demonstrated that LRRC8A is essential for VRAC activity and that it must be coexpressed with one of the other four *LRRC8* genes to reconstitute channel function. It is important to note, however, that two subsequent studies have suggested that LRRC8A may not be required for swelling‐activated anion currents observed in all cell types (Milenkovic et al. 2015; Sirianant et al. 2016).

Our findings confirm and extend the studies from the Jentsch and Patapoutian labs (Qiu et al. 2014; Voss et al. 2014; Syeda et al. 2016). We identified *LRRC8A* and *LRRC8C‐E* orthologs in the zebrafish genome. A *LRRC8B* ortholog was not identified. *LRRC8B* genes were likely lost from the genomes of ray‐finned fishes (Abascal and Zardoya 2012).

Zebrafish embryo cells express a robust swelling‐activated anion current with properties similar to those of VRAC currents observed in countless mammalian cell types. Knockdown of *lrrc8aa* expression with either translation or splice blocking morpholinos eliminates or dramatically reduces these currents (Fig. 4). Swelling‐activated anion currents are reconstituted in *LRRC8* ^*−/−*^ HCT116 cells coexpressing of *lrrc8aa* and human *LRRC8C* cDNAs (Fig. 5). We conclude that zebrafish express a bona fide VRAC and that the zebrafish *LRRC8A* ortholog *lrrc8aa* is essential for VRAC activity.

*LRRC8A* knockout mice exhibit increased in utero and postnatal mortality, growth retardation and abnormalities in multiple tissue types (Kumar et al. 2014). A patient with a heterozygous *LRRC8A* truncation mutation that likely functions as a dominant‐negative suppressor of LRRC8A function has been reported. This patient lacks B cells and has facial abnormalities and agammaglobulinemia (Sawada et al. 2003). *LRRC8C* has been proposed to play a role in adipocyte differentiation (Tominaga et al. 2004).

Consistent with these findings in humans and mice, we found that *lrrc8aa* is required for normal zebrafish development (Fig. 6). Indirect evidence suggests that VRAC activity may play a role cell differentiation, proliferation, migration and programmed cell death (reviewed in Mulligan and MacVicar 2006; Hoffmann et al. 2009; Duran et al. 2010; Pedersen et al. 2015), processes that are required for normal development.

With the discovery of the role of LRRC8 proteins in VRAC function, it should now be possible to begin defining the precise physiological functions of the channel beyond its well defined role in cell volume regulation. However, it will be important to determine whether a specific physiological process requires VRAC channel activity per se or another function of the LRRC8 proteins. Leucine‐rich repeat domains mediate diverse protein‐protein interactions (Kobe and Kajava 2001; Abascal and Zardoya 2012). It is thus conceivable that LRRC8 proteins serve a scaffold function to bring signaling and other components together that regulate a physiological process independently from VRAC activity. The amenability of zebrafish to detailed developmental, molecular and genetic analyses should make this model a valuable tool for defining the functional roles of LRRC8 proteins and the VRAC channel.

In summary, our studies have provided the first characterization of zebrafish VRAC activity and of the zebrafish *LRRC8A* ortholog. Our findings demonstrate that zebrafish *lrrc8aa* plays an essential role in VRAC function, provide an important confirmation of the recent work of the Jentsch and Patapoutian labs, and establishes the zebrafish as a genetically tractable model organism for the cellular and molecular characterization of *LRRC8* biology.

## Conflicts of Interest

The authors declare that they have no conflicts of interest with the contents of this article.
